# Sugar stress attenuates fruity aroma in sweet wine by suppressing ethyl ester biosynthesis: Insights from integrated sensory, metabolome, and transcriptomic analyses^☆^

**DOI:** 10.1016/j.fochx.2026.103580

**Published:** 2026-01-30

**Authors:** Ruyi Li, Wenzhe Tong, You Liu, Qian Ge, Xiaoyu Xu, Keji Yu, Wenyu Shi, Haibin Mu, Guoliang Yan, Changqing Duan, Yibin Lan

**Affiliations:** a Center for Viticulture and Enology, College of Food Science & Nutritional Engineering, China Agricultural University, Beijing 100083, China; b Key Laboratory of Viticulture and Enology, Ministry of Agriculture and Rural Affairs, Beijing 100083, China; c Institute of Quality Standard and Testing Technology for Agro-products of Ningxia, Yinchuan 750002, China; d MOA Key Laboratory of Soil Microbiology, College of Biological Sciences, China Agricultural University, Beijing 100193, China; e Collaborative Innovation Center for Wine Industry Technology of Ningxia Helan Mountain Eastern Foothills, Yinchuan 750104, China

**Keywords:** Sweet wine fermentation, Fruity aroma, *Saccharomyces cerevisiae*, Sugar stress, Ethyl ester biosynthesis, Metabolomics, Transcriptome

## Abstract

Sugar-induced stress is a key characteristic of sweet wine fermentation, yet its impact on sensory properties remains poorly understood. In particular, the molecular mechanisms behind the reduction of fruity aroma under high-sugar conditions are unclear. In this study, we conducted an integrated analysis combining sensory evaluation, metabolomics, and transcriptomics of wines fermented from grape musts with three initial sugar concentrations (204, 260, and 316 g/L). Sensory and metabolomic profiling revealed that higher initial sugar concentrations resulted in a diminished fruity aroma, which was associated with a reduction in ethyl ester content. Transcriptomic analysis identified differentially expressed genes (DEGs) involved in ethyl ester metabolism, while weighted gene co-expression network analysis (WGCNA) revealed 14 candidate hub genes potentially regulating ethyl ester production in *Saccharomyces cerevisiae*. These findings provide new insights into the molecular mechanisms underlying the reduction of fruity aroma under sugar stress and offer a theoretical basis for enhancing fruity aroma retention in sweet wine production

## Highlights

• Analyses on wine fermented with three sugar concentrations (204, 260, 316 g/L).
• High sugar stress reduced fruity aroma, mainly due to lower ethyl ester levels.
• Ethyl ester dynamics under different sugar concentrations in sweet wine.
• Transcriptomics revealed sugar stress-induced genes in the ethyl ester pathway.
• Key hub genes identified in ethyl ester formation under high-sugar stress.

## 1 Introduction

Sweet wines have been valued for their preservation properties since ancient Rome and the Middle Ages. Today, the production of high-quality sweet wines continues to grow, driven by evolving consumer preferences and a focus on enhancing their unique flavor profiles (Reboredo-Rodríguez et al., 2015). Grape musts used in winemaking typically contain 16–26% (*w*/*v*) sugar, but for sweet wines, the sugar concentration can reach as high as 30% or even 50% (w/v) (Erasmus et al., 2003). High sugar concentrations (≥25% w/v) induce osmotic stress in yeast, causing dehydration, contraction, and growth inhibition (Chen et al., 2023). Additionally, high sugar levels induce oxidative stress in *Saccharomyces cerevisiae* (*S. cerevisiae*), leading to the production of large quantities of reactive oxygen species (ROS) (Noti et al., 2015). These ROS can damage cellular macromolecules (e.g., lipids, proteins, and DNA) when their levels exceed the antioxidant capacity of the cell (Chen et al., 2023). Furthermore, when the concentration of extracellular glucose exceeds 0.15 g/L, the Crabtree effect may occur, where yeast ferments sugar into ethanol even under aerobic conditions (*S. cerevisiae* typically accumulates ethanol only under anaerobic conditions) (Crabtree, 1929; Verduyn et al., 1984). This phenomenon, known as overflow metabolism, is characterized by high glycolytic flux, increased carbon flow to fermentation by-products, and decreased flux through respiration, even in the presence of oxygen (Malina et al., 2021). In high-sugar stress environments, yeast activates complex adaptive programs, including the suspension of cell cycle processes, modulation of gene transcription and translation, and synthesis and accumulation of osmoprotectants such as glycerol, predominantly regulated by the HOG signaling pathway (Guan et al., 2017; Tatebayashi et al., 2020). Beyond its impact on yeast physiology and metabolism, sugar stress can also alter the synthesis of key aroma compounds, thereby influencing the sensory quality of sweet wines.

Aroma is a key parameter in evaluating wine quality, with fruity notes closely associated with the presence of volatile esters, particularly ethyl esters (Ling et al., 2022). Research shows that ethyl esters are produced through the reaction between fatty acyl-CoA and ethanol, catalyzed by acyltransferases encoded by the *EEB1* and *EHT1* genes (Swiegers and Pretorius, 2005). While ethyl ester production is influenced by the composition of the grape juice and the fermentation environment (Antalick et al., 2015), its regulation under high-sugar stress conditions remains poorly understood.

Moreover, previous studies have shown inconsistent results regarding the effects of sugar concentration on ethyl ester production. For instance, Lu et al. (2018) found that increasing sugar concentrations in mango juice from 17 to 30° Brix reduced ethyl ester production by yeast. Conversely, Martín-García et al. (2023) observed no significant differences in the production of most ethyl esters among three different wine yeasts fermenting grape juice with initial sugar concentrations of 220 g/L and 250 g/L. Houtman et al. (1980) noted that higher initial sugar content (18 to 24° Brix) of grape juice increased production of ethyl octanoate and ethyl decanoate in wine. Likely, these inconsistent results may be attributed to the differences in initial sugar concentration, fermentation environment, and yeast strains.

Despite advances in metabolomics and transcriptomics, few studies have systematically examined how sugar stress affects the expression of fruity aroma and its underlying molecular regulation during sweet wine fermentation. Integrative approaches—combining sensory profiling, omics analyses, and network-based gene mining—have proven powerful in decoding complex traits such as aroma synthesis, anthocyanin biosynthesis, and mycotoxin accumulation (Ding et al., 2021; Ge et al., 2024; Zhang et al., 2024). Among these, Weighted Gene Co-expression Network Analysis (WGCNA) offers a robust framework to assess gene module connectivity and their correlation with phenotypic traits, enabling the identification of hub genes that potentially regulate complex biological processes (Zhou et al., 2021).

In this study, we aimed to elucidate the sensory and molecular mechanisms underlying the loss of fruity aroma in sweet wines under sugar stress, with a particular focus on ethyl ester biosynthesis. By integrating sensory analysis, metabolomics, transcriptomics, and WGCNA, we identified key sugar stress–responsive genes and transcriptional regulators involved in ethyl ester production in *S. cerevisiae*. Our findings provide new insights into how sugar stress leads to the reduction of fruity aroma in sweet wines and offer a theoretical foundation for improving fruity aroma retention in sweet wine production.

## 2 Materials and methods

### 2.1 Reagents

Analytical-grade chemicals, including diammonium phosphate, glucose, sodium chloride, sodium hydroxide, glycerol, and 3,5-dinitrosalicylic acid, were all purchased from Beijing Solarbio Science & Technology Co., Ltd. (Beijing, China). Yeast extract peptone dextrose agar (YPD) was purchased from Beijing Aoboxing Bio-Tech Co., Ltd. (Beijing, China). HPLC-grade reagents, including ethanol (≥99.8%), glycerol (≥99%), and acetic acid (99%), were purchased from Shanghai McLean Biochemical Technology Co., Ltd. (Shanghai, China). Details of all standards of aroma compounds were shown in Supplementary Table S1.

### 2.2 Must preparation

‘Riesling’ grapes were harvested from a commercial vineyard in the Huaizhuo Basin region of China (40°21′N-115°32′E). The grape juice was obtained through pressing and then was added with 30 mg/L of pectinase (Lafazym extract, Laffort, France). The juice was clarified at 6 °C for 36 h, then collected and pasteurized at 65 °C for 30 min before fermentation. The basic parameters of the juice were recorded as follows: pH 3.64, total acidity 5.4 g/L, and sugar concentration 204 g/L.

### 2.3 Fermentation trials

Based on prior laboratory research, the sugar content of grapes from eastern China typically ranges between 180 and 220 g/L (Han et al., 2022), while those from western China exhibit sugar levels between 220 and 280 g/L (Li et al., 2014; Lu et al., 2023). Therefore, the selected sugar concentration range in this research can be considered representative of grapes from both regions. Glucose and 200 mg/L of diammonium phosphate were added to the clarified ‘Riesling’ juice to obtain three grape juices with different sugar concentrations (204 g/L, 260 g/L, and 316 g/L). Fermentation was conducted in 500 mL flasks containing 400 mL of pasteurized juice. The must was inoculated with 200 mg/L of commercial yeast EC1118 (Lallemand, Blagnac, France) (*S. cerevisiae*), and the fermentation temperature was controlled at 18 °C without shaking. Each treatment was performed with three biological replicates.

During alcoholic fermentation, 5 mL samples were collected daily to ascertain the concentrations of reducing sugars and yeast cell counts. Fermentation samples were taken at the exponential phase (sugar consumption: 60, 80, 100, 120, and 140 g/L), the stationary phase (sugar consumption: 160 and 180 g/L), and the termination point of fermentation (sugar consumption: 200 g/L). Afterward, the fermentation liquid was centrifuged at 8000 rpm for 5 min at 4 °C. The supernatant was stored at −20 °C for analysis of volatile compounds, ethanol, acetic acid, and glycerol. The yeast cells were flash-frozen in liquid nitrogen and stored at −80 °C for RNA extraction.

### 2.4 Determination of yeast cell counts, reducing sugar, and major metabolites

Plate counts were used to monitor the yeast growth dynamics during the fermentation process. Daily, 1 mL aliquots of each sample were diluted in sterile physiological saline and plated on YPD agar. The yeast colonies were counted after 3–5 days of incubation at 28 °C (Englezos et al., 2018). The concentration of reducing sugars was measured using dinitrosalicylic acid method (Başkan et al., 2016). Basic parameters of the wines, including glycerol, ethanol, and acetic acid, were analyzed using an Agilent 1200 HPLC system (Agilent Technologies, Santa Clara, CA, USA), fitted with a 300 mm × 7.8 mm ion-exchange column (Aminex HPX—87H, Bio-Rad Laboratories, Hercules, CA, USA). Before HPLC analysis, samples were filtered using a 0.22 μm membrane filter (Dikma Technologies, Lake Forest, CA, USA). The eluent was composed of 5 mM H_2_SO_4_, flowing at a rate of 0.6 mL/min. A refractive index detector was used to measure ethanol and glycerol at a column temperature of 45 °C, while acetic acid was detected using a UV detector at 214 nm with the column maintained at 60 °C. Evaluation of all samples was conducted in duplicate.

### 2.5 Analysis of volatile compounds

Volatile compounds were extracted using headspace solid phase micro-extraction (HS-SPME) with a 2 cm DVB/CAR/PDMS 50/30 μm SPME fiber (Supelco, Bellefonte, PA., USA) and analyzed using a gas chromatograph (Agilent 7890 GC) combined with a mass spectrometer (Agilent 5975 B). A total of 5 mL of the sample, 1.5 g of sodium chloride, and 10 μL of the internal standard (4-methyl-2-pentanol, 1.0 g/L) were placed in a 20 mL vial sealed by a PTFE‑silicon septum. After equilibration at 40 °C for 30 min in a CTC CombiPAL autosampler (CTC Analytics, Zwingen, Switzerland), the SPME fiber was inserted into the headspace of the vial to extract volatile compounds at the temperature of 40 °C for 30 min with stirring at 500 rpm. After that, the SPME fiber was thermally desorbed in the GC injector for 8 min at 250 °C with 5:1 split mode. Helium gas (>99.999% purity) was the carrier gas at a flow rate of 1 mL/min. The column used was an HP-INNOWAX (60 m × 0.25 mm × 0.25 μm, J&W Scientific, Folsom, CA, USA) with a temperature program starting at 50 °C for 1 min, increasing by 3 °C/min to 220 °C, and holding for 5 min. The mass spectrometer interface temperature was set at 250 °C, utilizing electron impact ionization (EI) mode at 70 eV, with an ion source temperature of 230 °C and a quadrupole temperature of 150 °C, scanning masses from 29 to 350 u. Each sample was analyzed in triplicate for technical replicates. For volatile compounds during wine fermentation, the relative peak area (RPA) values of each compound were determined as the peak area ratio of the identified compounds to the internal standard (4-methyl-2-pentanol). The loss rate of aroma compounds was calculated according to Zhang et al. (2022).

Loss rate (%) = 1-(the final RPA value/ the highest RPA value during fermentation) × 100%.

The concentrations of volatile compounds in the final wines were determined using calibration curves derived from standards of these compounds at different concentrations in a synthetic model wine solution (12% *v*/v ethanol, 2 g/L glucose, 7 g/L tartaric acid, pH 3.5).

### 2.6 Sensory analysis

#### 2.6.1 General conditions

Sensory evaluations were conducted in a clean, odor-free, and well-lit sensory laboratory at 20 °C, with each panelist having an individual booth and light source. Approximately 30 mL of wine was prepared in an International Standards Organization (ISO) wine tasting glass (ISO 3591:1977), coded with three-digit random numbers, and presented to panelists in random order.

#### 2.6.2 Panel training

The wine samples were assessed by a sensory panel consisting of eleven experienced members from the Centre for Viticulture and Enology at China Agricultural University (4 females and 7 males, aged 22–35). All panelists were selected based on their motivation and availability and were trained according to ISO 4121 (https://www.iso.org/standard/33817.html). They attended four training sessions over the course of one month (once a week, 2 h each) before the formal evaluation. In the first session, panelists were asked to evaluate all wine samples and generate descriptors. In the second and third sessions, reference standards representative of the descriptors were prepared. Further discussion followed based on the reference standards until everyone agreed on the descriptors. The reference standards used in the trained panel evaluations are listed in Supplementary Table S2. In the final session, all panelists were asked to familiarize themselves further with the targeted sensory attributes and score their intensities. Panel assessment was conducted during training sessions according to the method described by Lan et al. (2019).

#### 2.6.3 Quantitative descriptive analysis (QDA)

During training, all panelists agreed that ten aroma attributes (vegetal, floral, apple, pineapple, honey, peach, citrus, passion fruit, kerosene, and chemical) and overall aroma intensity could describe the aroma characteristics of wine samples. Additionally, panelists were required to rate the intensity of these attributes on an 10-point scale (0 = very low intensity, 10 = strong intensity).

### 2.7 RNA-seq analysis

Transcriptomic analyses were conducted on fermentation samples from yeast consuming 120 and 180 g/L of sugar, designated as L-EP, M-EP, H-EP, L-SP, M-SP, and H-SP. Here ‘L', ‘M', and ‘H' refer to fermentation samples with initial sugar concentrations of 204, 260, and 316 g/L, respectively. The samples L-EP, M-EP, and H-EP correspond to yeast consuming 120 g/L of sugar, whereas L-SP, M-SP, and H-SP correspond to yeast consuming 180 g/L of sugar. Total RNA was extracted from the *S. cerevisiae* using TRlzol Reagent (Thermo Fisher Science, Wilmington, USA). RNA concentration was determined using a NanoDrop 2000 (Thermo Fisher Scientific, Wilmington, DE, USA), and RNA integrity was assessed using the RNA Nano 6000 Assay Kit on the Agilent Bioanalyzer 2100 system (Agilent Technologies, Palo Alto, CA, USA). Sequencing libraries were generated using the Hieff NGS Ultima Dual-mode mRNA Library Prep Kit for Illumina (Yeasen Biotechnology, Shanghai, China) following the manufacturer's recommendations, and index codes were added to attribute sequences to each sample. The library preparations were sequenced on an Illumina NovaSeq 6000 platform (Illumina, Inc., San Diego, USA). The raw reads were further processed with the bioinformatic pipeline tool BMKCloud (https://www.biocloud.net).

Differential expression analysis of two groups was performed using edgeR (version 3.30.0) software. For identifying differentially expressed genes (DEGs), a fold change (FC) ≥ 1.5 and *p* < 0.05 were used as the screening criteria. FC represents the ratio of gene expression values in two groups. Gene function was annotated based on the following databases: Nr (NCBI non-redundant protein sequences), Pfam (Protein family), KOG/COG (Clusters of Orthologous Groups of proteins), Swiss-Prot (a manually annotated and reviewed protein sequence database), and GO (Gene Ontology). Kyoto Encyclopedia of Genes and Genomes (KEGG) pathway analysis was performed using the GENE DENOVO platform (https://www.omicshare.com/tools/Home/Soft/pathwaygsea), with differential genes significantly enriched in the pathways identified using a q-value threshold of <0.05.

### 2.8 Quantitative real-time PCR (qRT-PCR) analysis

Twelve DEGs were selected for qRT-PCR analysis to verify RNA-seq results. Total RNA was isolated using a HiPure Yeast & Microboil RNA Kit (Magen Biotechnology Inc., China). The RNA concentration was measured using a NanoDrop-2000 (Thermo Fisher Scientific Inc., Waltham, USA). First-strand cDNAs were synthesized by 1 μg total RNA samples using Hiscript® III RT SuperMix for qPCR (+gDNA wiper) (Vazyme, China.) in three biological replicates. Reactions were carried out on a real-time PCR system (CFX384, Bio-Rad, USA.) using SYBR master mix (Q711, Vazyme, China.). The *ACT1* gene of *S. cerevisiae* was selected as the reference gene, and the designed primer sequences are shown in Table S3. The average threshold cycle was calculated to estimate the relative gene expression levels using the 2^−ΔΔCt^ method (Livak & Schmittgen, 2001).

### 2.9 Weighted gene co-expression network analysis (WGCNA)

WGCNA of ethyl ester data from 18 samples was conducted using the WGCNA R package (v 1.68) (Langfelder & Horvath, 2008). Network construction and module detection were performed using an unsigned topological overlap matrix (TOM), with a power β of 14 and a module cuttree height of 0.25. The minimum number of genes per module was set to 30. Significant modules were selected based on two criteria: (1) significant correlation with ethyl ester levels and (2) more than half of the ethyl esters in a module showing a high correlation coefficient (≥ 0.8). KEGG pathway analysis was performed using the GENE DENOVO platform (https://www.omicshare.com/tools/Home/Soft/pathwaygsea). Regulation networks of hub genes were visualized using Cytoscape software (version 3.10.2).

### 2.10 Statistical analysis

All experimental data in this work are expressed as mean ± standard deviation (SD). SPSS version 22.0 (IBM Corporation, USA) was used for all significance analyses at *p* < 0.05 (Duncan's multiple range test) and Pearson's correlation analysis. Figures were generated by Origin 2022 (OriginLab, Northampton, Massachusetts, USA), SIMCA 14.1 (Umetrics, Malmo, Sweden), Cytoscape software (version 3.8.2), and TBtools (version 1.082, China).

## 3 Results and discussion

### 3.1 Evolution of yeast cell counts, reducing sugar, and major metabolites during fermentation

**Fig. 1:**
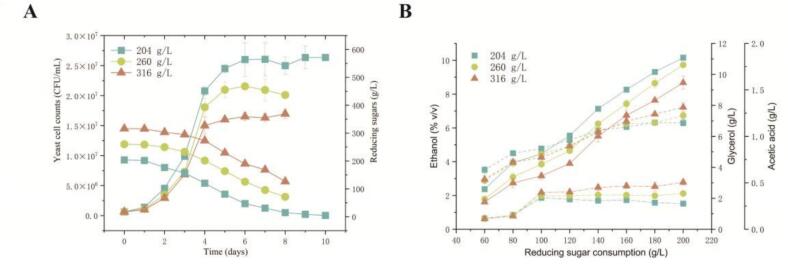
Dynamics of yeast cell counts, reducing sugar consumption (A), and major metabolites (ethanol, solid line; glycerol, dashed line; acetic acid, dotted line) (B) in wine alcohol fermentation under different initial concentrations of sugar. (204 g/L, green square, 260 g/L, yellow circle, 316 g/L: orange triangle). (For interpretation of the references to colour in this figure legend, the reader is referred to the web version of this article.)

Fig. 1A shows the colony growth of *S. cerevisiae* under different initial sugar concentrations. As the initial sugar concentration increased, the number of yeast colonies decreased, indicating the inhibitory effect of high sugar levels on yeast growth, consistent with the findings of Heit et al. (2018). Specifically, at initial sugar concentrations of 204 g/L, 260 g/L, and 316 g/L, yeast cell counts stabilized on the fifth day of fermentation, reaching 2.6 × 10^7^, 2.1 × 10^7^, and 1.6 × 10^7^ CFU/mL, respectively. Significant differences among these sugar levels suggest varying degrees of osmotic stress imposed on the yeast. The reduced growth rate under high-sugar conditions could be attributed to osmotic and oxidative stress, both of which are known to impede yeast metabolism and growth (Chen et al., 2023; Noti et al., 2015).

Regarding sugar consumption (Fig. 1A), during the first four days of fermentation, the rate of sugar consumption decreased as the initial sugar concentration increased. The initial delay in sugar consumption at higher sugar concentrations may reflect the adaptive process to osmotic stress, which temporarily reduces the yeast's metabolic activity. At sugar concentrations of 204 g/L, 260 g/L, and 316 g/L, yeast consumed 200 g/L of sugar by days 10, 8, and 8 of fermentation, respectively.

Fig. 1B shows the production of major metabolic products. Under all three initial sugar concentrations, the accumulation of ethanol, acetic acid, and glycerol by *S. cerevisiae* increased with the consumption of sugar. By the end of fermentation, yeast at the 316 g/L initial sugar concentration accumulated the highest levels of acetic acid and glycerol, reaching 0.51 g/L and 7.87 g/L, respectively, while ethanol accumulation was the lowest at 8.68 g/L. Glycerol, as a compatible solute, helps regulate intracellular osmotic pressure, protecting cells from osmotic stress (Lee & Oh, 2016), and its synthesis is accompanied by NAD+ generation. The formation of acetic acid helps to balance the excess NAD+ resulting from glycerol production, thereby maintaining cellular redox equilibrium (Pigeau & Inglis, 2005). Additionally, the concentration of glycerol decreased as the initial sugar concentration increased before 140 g/L of sugar was consumed. This trend likely reflects the yeast's physiological response to early osmotic stress during fermentation, where glycerol accumulates intracellularly to counteract external stress and is then secreted outside the cell as the yeast gradually adapts to the osmotic environment.

### 3.2 Sensory properties in final wines

**Fig. 2:**
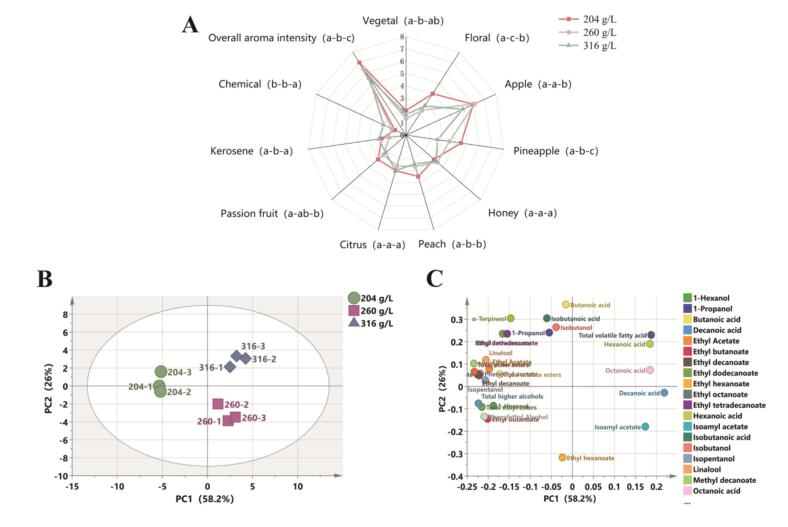
Sensory analysis (A) was conducted to assess the differences between wines produced with varying sugar concentrations. A principal component analysis (PCA) score plot (B) and loadings plot (C) were constructed based on the volatile compound contents.

The specific sensory analysis results are shown in Fig. 2A. As the initial sugar concentration increased, the overall aroma intensity of the wine decreased, with significant differences observed among the samples. Wine fermented with an initial sugar concentration of 204 g/L displayed stronger floral, apple, pineapple, peach, and passion fruit aromas. In contrast, wine fermented with an initial sugar concentration of 316 g/L exhibited weaker fruity aromas. Additionally, it displayed stronger chemical notes—considered “unpleasant” in wine—such as rancid, pungent, and synthetic aromas (Ríos-Reina et al., 2020).

Overall, wines fermented at higher initial sugar concentrations showed a reduction in fruity aromas and overall aroma intensity.

### 3.3 Volatile compounds in final wines

To further investigate the causes of sensory differences in wine after fermentation at different initial sugar concentrations, the aroma components of the wine were analyzed. A total of 42 aromatic compounds were identified, including 20 esters, 8 higher alcohols, 4 C_6_ alcohols, 6 volatile fatty acids, and 4 other aromatic compounds in the final wines (Table 1). Among these, 22 compounds had odor activity values (OAV) greater than 0.1, indicating their potential contribution to the odor of the wine (Gómez García-Carpintero et al., 2012). Acetate esters, ethyl esters, higher alcohols, and volatile fatty acids were identified as the primary fermentation aromas in the wine, playing a significant role in shaping the overall aromatic profile.

**Table 1:** The contents of volatile compounds in final wines fermented by different initial concentrations of sugar.

Compounds	Concentration (μg/L) a			Threshold (μg/L) b	Odor description
	204 g/L	260 g/L	316 g/L		
Esters					
Isobutyl acetate	18.68 ± 2.50a	19.16 ± 0.68a	20.34 ± 0.23a	1600[1]	Fruty, apple
Isoamyl acetate	**976.47** **±** **9.61b**	**1040.20** **±** **16.09a**	**1005.59** **±** **23.56ab**	160 [2]	Banana
Hexyl acetate	18.33 ± 0.06a	18.74 ± 0.14a	16.52 ± 0.27b	670 [2]	Apple, cherry, pear, floral
Heptyl acetate	0.04 ± 0.00a	0.04 ± 0.00a	0.04 ± 0.01a		Almond, pear
Phenethyl acetate	**184.13** **±** **0.57a**	164.48 ± 2.89b	164.78 ± 4.15b	1800 [2]	Fruity, rose
Ethyl acetate	**30,234.55** **±** **597.34a**	**28,315.17** **±** **1392.63b**	**28,280.31** **±** **114.41b**	7500 [3]	Pineapple, solvent
Total acetate esters	31,039.21 ± 1067.11a	29,695.72 ± 360.79a	29,487.58 ± 142.62a		
Ethyl butanoate	**148.25** **±** **1.83a**	**127.59** **±** **3.62b**	**104.07** **±** **1.53c**	400 [2]	Banana, pineapple, strawberry
Ethyl hexanoate	**469.81** **±** **7.82b**	**529.11** **±** **9.11a**	**423.29** **±** **11.55c**	80 [2]	Banana, green apple
Ethyl (*E*)-3-hexenoate	0.09 ± 0.00a	0.09 ± 0.00a	0.07 ± 0.00b		
Ethyl octanoate	**859.91** **±** **18.03a**	**447.92** **±** **11.79c**	**490.53** **±** **6.75b**	580 [2]	Sweet, floral, banana, pear
Ethyl decanoate	**1323.43** **±** **62.81a**	**903.69** **±** **21.88b**	**951.38** **±** **36.97b**	200 [4]	Fruity, fatty
Ethyl 9-decenoate	5.05 ± 0.16a	2.50 ± 0.04b	2.46 ± 0.23b	100 [5]	Fatty
Ethyl dodecanoate	**892.13** **±** **0.89a**	**767.49** **±** **54.12b**	**858.63** **±** **4.24ab**	1500 [6]	Fruity，fatty
Ethyl tetradecanoate	**73.93** **±** **1.24a**	**65.28** **±** **0.23b**	**72.06** **±** **0.81a**	500 [7]	Mild waxy, soapy
Ethyl hexadecanoate	**153.12** **±** **3.20a**	135.75 ± 6.49a	148.40 ± 14.81a	1500 [6]	Mild waxy
Total ethyl esters	3956.92 ± 67.99a	2946.17 ± 89.04b	2277.93 ± 158.10c		
Methyl hexanoate	1.28 ± 0.01b	1.68 ± 0.04a	1.23 ± 0.02b		
Methyl octanoate	1.99 ± 0.00a	1.44 ± 0.07b	1.40 ± 0.02b	100 [8]	Intense citrus
Isobutyl octanoate	2.27 ± 0.02a	2.14 ± 0.00b	2.16 ± 0.01b		
Methyl decanoate	**131.47** **±** **1.71a**	90.92 ± 5.59b	98.93 ± 1.22b	1200 [8]	Waxy, soap, fruity
Isoamyl octanoate	8.93 ± 0.11a	5.22 ± 0.25c	6.00 ± 0.02b	125 [4]	Sweet, fruity, cheese, cream
Total other esters	144.17 ± 0.90a	104.40 ± 4.03b	106.74 ± 5.46b		
Higher alcohols					
1-Octanol	3.15 ± 0.18a	2.22 ± 0.50b	1.68 ± 0.36b	800 [9]	Jasmine, lemon
Isopentanol	**106,223.86** **±** **4278.65a**	**91,648.60** **±** **139.61b**	**91,648.60** **±** **139.61b**	60,000 [2]	Solvent, alcohol, nail polish
1-Propanol	**48,796.20** **±** **2863.79a**	**46,316.22** **±** **1518.94a**	**48,595.05** **±** **1447.36a**	306,000 [2]	Alcohol, ripe fruit
1-Butanol	331.74 ± 12.81a	375.90 ± 29.59a	356.86 ± 29.45a	150,000 [2]	Medicinal, phenolic
Isobutanol	**11,295.49** **±** **863.48a**	**11,295.49** **±** **863.48a**	**11,439.94** **±** **369.42a**	75,000 [2]	Alcohol, solvent, green, bitter
4-Methyl-1-pentanol	175.07 ± 3.50a	150.92 ± 7.15b	161.06 ± 4.99ab	50,000 [10]	Almond, toasted
1-Decanol	2.64 ± 0.13a	1.89 ± 0.03b	1.83 ± 0.09b	400 [4]	Orange flowery, special fatty
2-Phenylethyl alcohol	**12,689.20** **±** **80.40a**	**11,389.71** **±** **362.56b**	**10,192.41** **±** **356.88c**	14,000 [4]	Rose, honey
Total higher alcohols	170,425.66 ± 737.22a	160,259.17 ± 688.79b	156,285.51 ± 3022.08b		
C_6_ alcohols					
1-Hexanol	**982.99** **±** **77.96a**	**861.70** **±** **29.65ab**	**803.66** **±** **9.03b**	1100 [2]	Herbaceous,grass, woody
(*E*)-2-Hexen-1-ol	7.81 ± 0.03a	6.46 ± 0.32b	6.85 ± 0.57ab	15,000 [11]	Herbaceous,grass
(*Z*)-3-Hexen-1-ol	54.19 ± 0.80a	53.93 ± 1.45a	49.91 ± 0.38b	1000 [2]	Herbaceous, grass
(*E*)-3-Hexen-1-ol	19.68 ± 3.10a	20.90 ± 1.21a	17.79 ± 1.85a	1000 [11]	Herbaceous, grass
Total C_6_ alcohols	76.18 ± 4.23a	78.18 ± 4.49a	75.37 ± 2.32a		
Volatile fatty acids					
Hexanoic acid	**2087.23** **±** **141.75b**	**2271.97** **±** **87.32b**	**2636.25** **±** **69.10a**	420 [4]	Cheese, fatty
Octanoic acid	**7745.35** **±** **292.11a**	**8332.76** **±** **260.46a**	**8589.89** **±** **719.64a**	500 [4]	Rancid, cheese, fatty acid
Decanoic acid	**5951.72** **±** **370.79b**	**7349.16** **±** **46.39a**	**7275.15** **±** **423.72a**	1000 [4]	Fatty, rancid
Butanoic acid	**594.52** **±** **24.28b**	**448.97** **±** **26.49c**	**657.30** **±** **4.55a**	2500 [7]	Cheese, rancid
Isopentanoic acid	**106.57** **±** **6.88a**	**101.13** **±** **4.91a**	**102.45** **±** **3.80a**	33.4 [4]	Sweat, acid, rancid
Isobutanoic acid	**387.25** **±** **3.35a**	**345.79** **±** **11.74b**	**391.18** **±** **7.52a**	2300 [4]	Rancid, butter
Total volatile fatty acid	16,965.57 ± 761.49b	18,589.33 ± 298.89ab	20,117.99 ± 988.13a		
Others					
Citronellol	8.17 ± 0.23a	4.46 ± 0.17c	5.53 ± 0.11b	100 [9]	Rose
α-Terpineol	**141.52** **±** **2.61a**	**89.72** **±** **4.53c**	**126.77** **±** **0.96b**	250 [4]	Lilac
Linalool	**317.24** **±** **8.05a**	**225. 86b**	**251.87** **±** **11.52b**	25 [8]	Flowery, fruity, muscat
Styrene	0.82 ± 0.01a	0.80 ± 0.03a	0.80 ± 0.00a		Balsamic, gasoline

Blank cells indicate no available threshold.

a The data are presented as the mean ± standard deviation (μg/L) of concentrations (*n* = 3). Different letters in the same row indicate significant differences by Duncan's multiple range test (*P* < 0.05), and the compounds with odor activity values (OAV) > 0.1 are highlighted.

b [1] Sumby et al. (2010): determined in beer. [2] Peinado et al. (2004): determined in a 10% (*v*/v) ethanol solution adjusted to pH 3.5 with tartaric acid. [3] Guth (1997): determined in water/ethanol (90 + 10, *w*/w). [4] Ferreira, Lo'pez, & Cacho (2000): determined in a 11% water/ethanol solution containing 7 g/L glycerol and 5 g/L tartaric acid, pH adjusted to 3.4 with 1 M NaOH. [5] Tao and Zhang (2010): determined in a 9.72 g/100 g ethanol/water mixture containing 5 g/L tartaric acid at pH 3.2. [6] Li (2006). [7] Moyano et al. (2002): determined in a 14% (v/v) ethanol solution adjusted to pH 3.5 with tartaric acid. [8] Peng et al. (2013): determined in a 12% ethanol/water mixture containing 5 g/L tartaric acid at pH 3.2; [9] Peinado et al. (2006): determined in a 10% (v/v) ethanol–water solution, adjusted to pH 3.5 with tartaric acid. [10] Zea, L., Moyano, L., Moreno, J. A., & Medina, M (2007): determined in a 14%(v/v) ethanol solution; [11] Franco et al. (2004): determined in a 1:10 alcohol/water mixture.

Wine contains two primary types of esters: ethyl esters of fatty acids and higher alcohol acetates, both of which contribute to the fruity aromas in wine (Longo et al., 2021). As shown in Table 1, the total amounts of acetate esters and ethyl esters in the wine decreased with increasing sugar concentration, which was consistent with previous research findings (Lu et al., 2018). Specifically, compared to the 204 g/L initial sugar concentration, the total amounts of acetates and ethyl esters were reduced by 4.32% and 4.99% at 260 g/L, and by 25.51% and 42.42% at 316 g/L. Further analysis revealed that isoamyl acetate, phenethyl acetate, and ethyl acetate were the predominant acetates, accounting for over 95% of the total acetates. The levels of ethyl acetate and phenethyl acetate significantly decreased with increasing sugar concentration, with reductions of 6.32% and 6.45% at 260 g/L, and 10.67% and 10.51% at 316 g/L compared to 204 g/L. Additionally, ethyl butyrate, ethyl hexanoate, ethyl octanoate, ethyl decanoate, and ethyl dodecanoate were the primary ethyl esters, with ethyl octanoate and ethyl decanoate showing significant decreases as sugar concentration increased. Compared to the control, ethyl octanoate and ethyl decanoate levels decreased by 47.91% and 42.96% at 260 g/L, and by 31.72% and 28.11% at 316 g/L.

Higher alcohols are generated by yeast through the metabolism of sugars or amino acids. The results indicate that the total amount of higher alcohols decreased with increasing sugar concentrations (Table 1), consistent with the findings of Lu et al. (2018). Compared to the 204 g/L initial sugar concentration, higher alcohol levels decreased by 5.97% at 260 g/L and by 8.3% at 316 g/L. The primary higher alcohols detected in this study were isopentanol, 1-propanol, isobutanol, and 2-phenylethyl alcohol. Among these, the concentrations of isopentanol and 2-phenylethyl alcohol significantly declined with increasing sugar concentration, with reductions of 13.72% and 14.71% at 260 g/L and 10.24% and 19.70% at 316 g/L compared to the 204 g/L concentration.

Fatty acids are generally considered to negatively impact on wine quality, being associated with rancid, buttery, and cheesy odors (Liu et al., 2023). The results suggest that higher initial sugar concentrations promote the production of volatile fatty acids. Compared to the 204 g/L initial sugar concentration, the total amount of volatile fatty acids increased by 22.12% at 260 g/L and by 65.94% at 316 g/L. The primary volatile fatty acids detected in this study were hexanoic acid, octanoic acid, and decanoic acid. Among these, the levels of hexanoic acid and decanoic acid significantly increased with higher sugar concentrations. Specifically, compared to the initial sugar concentration of 204 g/L, the concentrations of hexanoic acid and decanoic acid showed an increase of 8.85% and 26.30% at 260 g/L, and 23.53% and 18.27% at 316 g/L, respectively.

The results of the principal component analysis (PCA) of aroma compounds in the wine after fermentation are presented in Fig. 2B and Fig. 2C. The two principal components accounted for 84.2% of the total variance, with PC1 explaining 58.2% and PC2 explaining 26%. PC2 distinguished fermentation samples with different initial sugar concentrations. Samples with a 204 g/L initial sugar concentration were positioned at the origin of the y-axis, associated with higher levels of ethyl butanoate, ethyl octanoate, ethyl decanoate, phenethyl acetate, 2-phenylethyl alcohol, isopentanol, and 1-hexanol. Samples fermented with 260 g/L initial sugar concentration were positioned on the negative side of the y-axis and linked to higher concentrations of ethyl hexanoate and isoamyl acetate. Meanwhile, the samples with a 316 g/L initial sugar concentration were situated on the positive side of the y-axis, closely associated with hexanoic acid and octanoic acid.

Through our analysis of the aroma at fermentation termination, we found that the total ethyl ester content, which contributes to fruity aromas, significantly decreased with increasing sugar concentration. This decrease directly correlates with the weakening of fruity aromas, further indicating that the total ethyl ester content is a critical aroma compound influencing the loss of fruity notes in sweet wines with higher sugar concentrations.

### 3.4 Dynamic analysis of ethyl ester during wine fermentation

During wine fermentation, the formation and degradation of aromatic compounds is a dynamic and complex process. Monitoring the changes in ethyl esters throughout fermentation provides a deeper understanding of their potential impact on the overall flavor profile of the wine and helps elucidate the reasons behind the variations in ethyl ester accumulation under different sugar stress conditions. Moreover, the continuous production and accumulation of ethanol during fermentation lead to significant changes in its concentration, which affect the detection of volatile compounds (Liu et al., 2020). To reflect the evolution of ethyl esters during fermentation, this study employed the RPA between ethyl esters and internal standards as a quantification method, which has been widely used in previous research (Lee et al., 2010; Zhang et al., 2022).

**Fig. 3:**
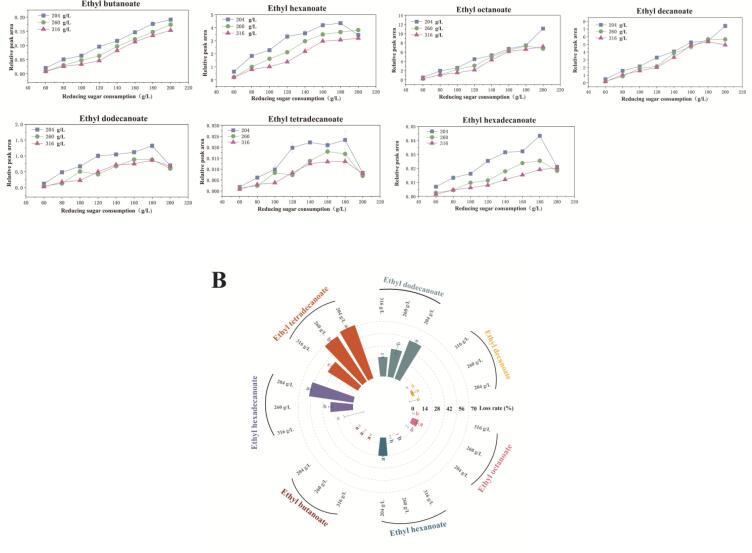
The change in RPA values (A) and the loss ratio (B) of ethyl esters in wine produced with different concentrations of sugar.

Fig. 3A illustrates the evolution of the ethyl ester across different initial sugar concentrations (204, 260, and 316 g/L). As fermentation progressed, the accumulation of yeast-derived esters (except for ethyl dodecanoate, ethyl tetradecanoate, and ethyl hexadecanoate) steadily increased with sugar consumption, with a notable acceleration observed during the later stages of exponential growth (approximately 120 g/L sugar consumption). Furthermore, a higher initial sugar concentration resulted in reduced ethyl ester accumulation. Analysis of ester loss (Fig. 3B) revealed that long-chain fatty acid ethyl esters, particularly ethyl dodecanoate, ethyl tetradecanoate, and ethyl hexadecanoate, were the most unstable and exhibited the greatest losses, whereas short- and medium-chain esters were comparatively less affected. As reported by Saerens et al. (2010), the proportion of fatty acid ethyl esters secreted outside the cell decreases with increasing carbon chain length, which may explain the higher loss ratios observed for ethyl dodecanoate, ethyl tetradecanoate, and ethyl hexadecanoate.

In summary, sugar stress not only limited the synthesis of most ethyl esters but also increased the degradation of long-chain fatty acid ethyl esters. The most pronounced differences appeared when yeast consumed 120 g/L and 180 g/L of sugar, suggesting that adaptation to a high-osmotic environment during these critical stages may substantially reprogram yeast metabolism, thereby shaping the final wine aroma.

### 3.5 Transcriptome analysis

Previous research observed that the accumulation of ethyl esters in wine showed the greatest difference at two distinct growth phases of yeast, the exponential and stationary phases, when sugar consumption was 120 g/L and 180 g/L, respectively. We performed transcriptomic analysis on samples from these two sugar consumption points to further investigate the potential molecular mechanisms underlying the influence of sugar concentration on ethyl ester production by *S. cerevisiae*. For convenience, we used low sugar (L), medium sugar (M), and high sugar (H) to represent samples with initial sugar concentrations of 204 g/L, 260 g/L, and 316 g/L, respectively. Additionally, we designated the samples with sugar consumption levels of 120 g/L and 180 g/L as the exponential phase (EP) and stationary phase (SP), respectively.

A total of 124.41 Gb of clean data was obtained after mRNA sequencing of 18 samples, each containing at least 6.31 Gb of clean data. In each sample, more than 95.84% had base scores ≥ Q30 (indicating sequencing error rates <0.1%), reflecting the high quality of the obtained bases. The clean data were then mapped to th1e *S. cerevisiae* genome, with the mapping ratio varying from 96.73% to 98.06%. The GC (Guanine-Cytosine) percentages ranged from 41.31% to 41.89% (Table S4). These data showed that RNA-seq data were of high quality and could be used for further analysis.

To validate the accuracy and reproducibility of the transcriptome analysis results, 12 genes were randomly selected and confirmed by qRT-PCR. The results showed that the qRT-PCR data, which proved were consistent with the trend of expression levels detected by RNA-seq, confirmed the data were reliable (Fig. S1).

#### 3.5.1 DEGs analysis

To verify the reliability of the experiment and the appropriateness of sample selection, correlation analysis and PCA were conducted on the gene expression levels of the 18 samples, using Fragments Per Kilobase of transcript per Million mapped reads (FPKM) as the metric of gene expression. The correlation analysis (Fig. S2A) revealed that the correlation coefficients among the three biological replicates for each sugar concentration treatment were ≥ 0.9, indicating strong consistency. Furthermore, the PCA confirmed the clustering of biological replicates across different treatments (Fig. S2B). Thus, all samples were considered suitable for further analysis.

**Fig. 4:**
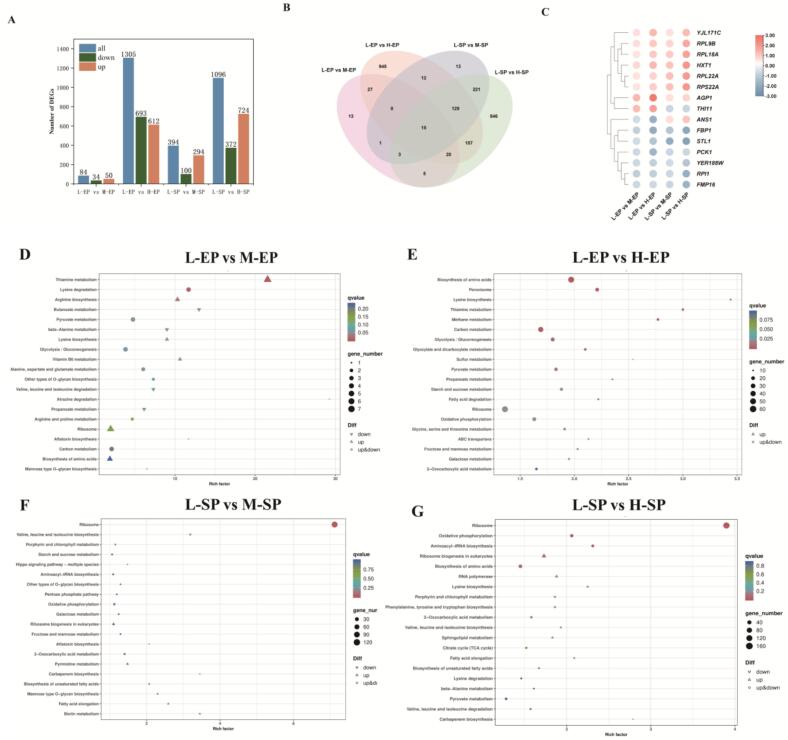
Transcriptome analysis in 18 samples**.** (A) The number of DEGs for different comparisons. (B) Venn diagram comparing significantly different expressed genes in different comparisons (C) The log_2_ FC value of common DEGs in four comparisons. (D-G) KEGG enrichment analysis of DEGs in different comparisons.

Further analysis of the number of DEGs among different treatments (Fig. 4A and Fig. S2C—F) revealed that 84 (50 upregulated, 34 downregulated), 1305 (612 upregulated, 693 downregulated), 394 (294 upregulated, 100 downregulated), and 1096 (724 upregulated, 372 downregulated) DEGs were identified in the comparisons of L-EP vs M-EP, L-EP vs H-EP, L-SP vs M-SP, and L-SP vs H-SP, respectively. The highest number of DEGs was observed in the comparisons of L-EP vs H-EP and L-SP vs H-SP, which showed that the H treatment had a much stronger effect on the transcriptome of the *S. cerevisiae* than M at both time points. Interestingly, in the L-EP vs H-EP comparison, the number of downregulated genes exceeded the number of upregulated ones, while the opposite was observed in L-SP vs H-SP, implying that high sugar concentrations may inhibit various physiological processes in *S. cerevisiae* during the early stages of fermentation.

The Venn diagram shows that 15 DEGs were common across all treatments (Fig. 4B-C and Table S5), with six of them being downregulated (*PCK1*, *FBP1*, *STL1*, *RPI1*, *FMP16*, and *YER188W*). Among the downregulated DEGs, both *PCK1* and *FBP1* are genes encoding enzymes in the gluconeogenesis pathway; *STL1* encodes the glycerol proton symporter, and *RPI1*, a transcription factor, mediates stress tolerance during fermentation by regulating cell wall integrity. The downregulation of *RPI1* is linked to a significant reduction in yeast viability (Puria et al., 2009). Under hyperosmotic conditions, *S. cerevisiae* requires antioxidants to scavenge ROS. *FMP16*, a mitochondrial protein, has a potential role in the stress response of mitochondria lacking respiratory chain complex II (Dannenmaier et al., 2018). Overexpression of *FMP16* has been shown to decrease ROS levels in the cytoplasm, mitochondria, and nucleus by 42.4%, 17.6%, and 24.4%, respectively (Liu, Lou, et al., 2023). However, its downregulation under hyperosmotic stress suggests the involvement of a more complex mechanism, which requires further investigation. Additionally, seven genes were consistently upregulated, primarily associated with glucose transport (*HXT1*), ribosomal proteins (*RPL9B, RPL22A, RPL18A,* and *RPS22A*), maintenance of cell wall integrity (*YJL171C*), and amino acid transport (*AGP1*). *YJL171C* contributes to maintaining cell integrity by participating in cell wall reorganization and repair in response to damage (García et al., 2004). These findings suggest that under hyperosmotic conditions, yeast cell wall integrity may be at risk, leading to the activation of repair mechanisms to preserve structural integrity. Overall, these shared DEGs play a crucial role in the yeast's response to osmotic stress during fermentation.

#### 3.5.2 KEGG enrichment analysis of DEGs

To elucidate the biological functions of these DEGs, KEGG enrichment analyses were conducted. The results revealed that 43, 106, 57, and 100 KEGG pathways were enriched in the comparisons of L-EP vs M-EP, L-EP vs H-EP, L-SP vs M-SP, and L-SP vs H-SP, respectively. Among these, 3, 19, 1, and 4 pathways were significantly enriched (*p* < 0.05) (Table S6–9). In the L-EP vs. M-EP comparison, DEGs were significantly enriched in pathways related to amino acid metabolism, including lysine degradation (ko00310) and arginine biosynthesis (ko00220), with all these genes showing an upregulation trend, indicating active protein synthesis in *S. cerevisiae* at this stage. In the L-EP vs H-EP comparison, the significantly enriched pathways included carbon metabolism (ko01200), glycolysis/gluconeogenesis (ko00010), pyruvate metabolism (ko00620), and fatty acid degradation (ko00071), all of which are closely related to ethyl ester synthesis. In L-SP vs M-SP, the DEGs were significantly enriched only in the ribosome pathway (ko03010). In L-SP vs H-SP, the DEGs were significantly enriched in pathways related to the ribosome (ko03010), oxidative phosphorylation (ko00190), aminoacyl-tRNA biosynthesis (ko00970), and ribosome biogenesis in eukaryotes (ko03008) (Fig. 4D-G).

Overall, during different growth stages of yeast, the H treatment significantly enriched more metabolic pathways compared to the M treatment. This could be attributed to the higher osmotic pressure faced by the yeast in the high-sugar environment, which requires broader metabolic adaptation. Additionally, the comparison between L-EP and H-EP revealed a significant enrichment of metabolic pathways closely related to ester synthesis. This finding further explains the differences in ethyl ester accumulation in wine when sugar consumption reaches 120 g/L.

#### 3.5.3 Sugar stress-induced gene expression profiles in the ethyl ester pathway

The synthesis of ethyl esters involves a series of biochemical reactions, including glycolysis, the pentose phosphate pathway, glycerol synthesis, the tricarboxylic acid (TCA) cycle, ethyl acetate synthesis, higher alcohol synthesis, and fatty acid synthesis. To further investigate the mechanism underlying ethyl ester accumulation in *S. cerevisiae*, the expression patterns of genes involved in the ethyl ester biosynthesis pathway were analyzed.

**Fig. 5:**
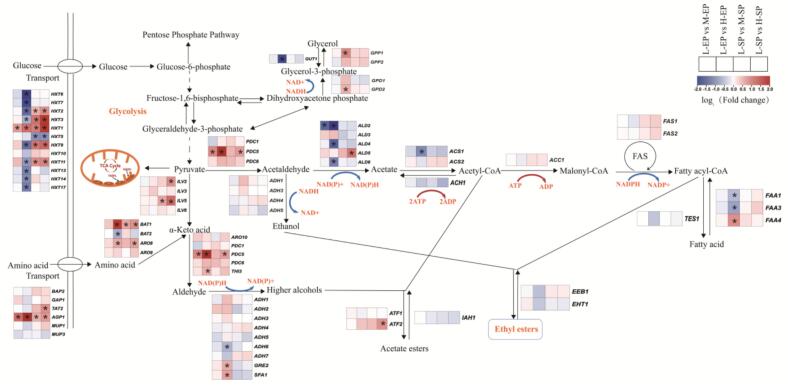
Biosynthetic pathways of ethyl ester in *S. cerevisiae*.

Glucose is a key substrate in the fermentation process of *S. cerevisiae*, and its uptake depends on sugar transporters encoded by the *HXT* genes. These transporters vary in their affinity for glucose, ranked from highest to lowest as *HXT6* > *HXT7* > *HXT2* > *HXT4* > *HXT3* > *HXT1* (Maier et al., 2002). During the exponential phase, high-sugar conditions led to the downregulation of high-affinity sugar transporters and the upregulation of low-affinity transporters (Fig. 5), consistent with previous findings (Jiménez-Martí et al., 2011). Furthermore, key hexokinases in the glycolysis, including *GLK1*, *HXK1*, and *YLR446W*, which are critical enzymes closely associated with ATP consumption, were downregulated under high-sugar conditions (Fig. S3). This regulation may prevent excessive ATP depletion during the initial steps of glycolysis, thereby enhancing yeast survival in high-sugar environments. Overall, most genes in the glycolytic pathway were significantly upregulated during the exponential phase under high-sugar treatment (Fig. S3). As a direct and efficient energy supply pathway (Deng, Du, Lu, Wang, and He, 2023), the increased glycolytic flux helps yeast cells rapidly respond to the energy demands associated with osmotic stress while also facilitating quick glucose consumption to alleviate environmental pressure.

Dihydroxyacetone phosphate, an intermediate of glycolysis, can be further converted into glycerol through the catalytic actions of glycerol-3-phosphate dehydrogenase (*GPD1,2*) and glycerol-3-phosphate phosphatase (*GPP1,2*) (Toledano et al., 2003). During the exponential phase, the expression of these enzyme-encoding genes was significantly upregulated under high-sugar conditions (Fig. 5), facilitating increased glycerol production to counteract the hyperosmotic environment. However, during the stationary phase, the expression of these genes decreased, indicating that the yeast had likely adapted to the high-osmotic environment, thereby decreasing the demand for glycerol production.

Additionally, the glycolytic intermediate glucose-6-phosphate can enter the pentose phosphate pathway for further metabolism. The oxidative branch of this pathway involves the generation of NADPH, which is crucial for yeast cells. Under high-sugar conditions, the expression of *SOL3* was significantly upregulated, which contributed to an increase in NADPH production (Fig. S4). NADPH is essential not only for directly neutralizing reactive oxygen species (ROS) but also as a key redox cofactor in the synthesis of glutathione and other antioxidant systems, such as thioredoxin-dependent enzymes, which help yeast cope with oxidative stress (Krüger et al., 2011; Perl et al., 2011).

Pyruvate, the end product of glycolysis, can be transported into mitochondria to participate in the TCA cycle. The results suggested that under high-sugar conditions, genes associated with the TCA cycle and the mitochondrial electron transport chain were predominantly downregulated (Fig. S5). The TCA cycle not only connects carbohydrate, protein, and fat metabolism (Baldwin & Krebs, 1981) but is also the hub of cellular energy metabolism (Liu, Qin, et al., 2023). The inhibition of TCA may be due to several factors: on the one hand, the accumulation of fructose-1,6-bisphosphate in the glycolysis pathway might intensify the suppression of complexes III and IV in the electron transport chain, leading to the accumulation of mitochondrial NADH and FADH_2_ (Lemus et al., 2018), which in turn suppresses the expression of TCA cycle-related enzymes. On the other hand, electron leakage from the electron transport chain (ETC) is considered a primary source of ROS in eukaryotic cells (Chevtzoff et al., 2010; Duncan et al., 2023). The downregulation of ETC and TCA activity in yeast cells may effectively limit ROS accumulation, mitigate oxidative stress-related damage, and improve their survival under environmental stress.

Finally, pyruvate is converted into ethyl ester through secondary metabolic pathways. It was found that aldehyde dehydrogenases (*ALD2, ALD4,* and *ALD6*) and acetyl-CoA synthetase (*ACS1*) were significantly downregulated in yeast under high-sugar conditions during the exponential phase. This downregulation may reduce the availability of precursors for ethyl ester biosynthesis, thereby impacting its production.

In general, yeast significantly upregulated the expression of genes related to glycolysis, the pentose phosphate pathway, glycerol synthesis, and low-affinity transporters under high-sugar conditions during the exponential phase. Conversely, the expression of genes associated with the TCA cycle, electron transport chain, and high-affinity transporters was downregulated. These regulatory mechanisms may enable yeast to better adapt to environments with high-sugar pressure, resulting in varying levels of ethyl ester accumulation during fermentation at different initial sugar concentrations.

### 3.6 Co-expression network analysis identified hub genes associated with ethyl ester synthesis

**Fig. 6:**
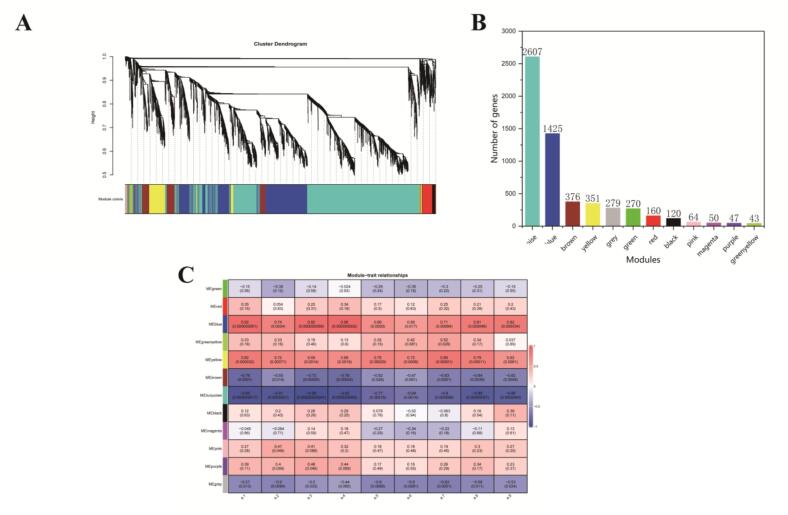

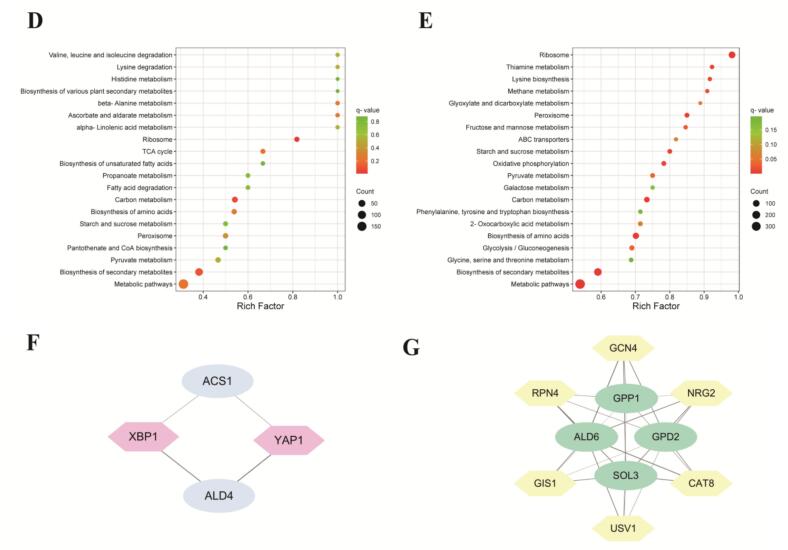
Co-expression network analysis. (A) A hierarchical clustering tree displayed 12 modules of co-expressed genes by weighted gene co-expression network analysis (WGCNA). (B) Number of genes in each module. (C) Module-trait relationships. Each row represents a module, and each column represents a specific trait. The compounds a1 to a9 correspond to ethyl butanoate, ethyl hexanoate, ethyl octanoate, ethyl decanoate, ethyl dodecanoate, ethyl tetradecanoate, ethyl hexadecanoate, ethyl (*E*)-3-hexenoate, and ethyl 9-decenoate. The colour scale on the right shows module-trait correlation from −1 (blue) to 1 (red). (D-E) KEGG enrichment analysis of DEGs in blue and turquoise modules. (For interpretation of the references to colour in this figure legend, the reader is referred to the web version of this article.)

To identify genes associated with ethyl ester accumulation under varying sugar stress conditions, a weighted gene co-expression network analysis (WGCNA) was conducted on 5792 genes with FPKM >1. The WGCNA generated 12 modules via the dynamic hybrid tree cut algorithm, namely turquoise, blue, brown, yellow, grey, green, red, black, pink, magenta, purple, and green-yellow, possessing 2607, 1425, 376, 351, 279, 270, 160, 120, 64, 50, 47, and 43 genes, respectively (Fig. 6A-B). Since gene expression patterns are often correlated with phenotypic changes, we analyzed the correlation between the expression patterns of each module and the variation in ethyl ester levels, with the results depicted in Fig. 6C. The results showed that the blue module exhibited a highly significant positive correlation with ethyl butanoate, ethyl octanoate, ethyl decanoate, ethyl (*E*)-3-hexenoate, and ethyl 9-decenoate (correlation coefficient ≥ 0.8, *P* < 0.05), while the turquoise module showed a highly significant negative correlation with ethyl butanoate, ethyl hexanoate, ethyl octanoate, ethyl decanoate, ethyl hexadecanoate, ethyl (*E*)-3-hexenoate, and ethyl 9-decenoate (correlation coefficient ≤ −0.8, *P* < 0.05). These two modules were thus selected for further analysis.

KEGG enrichment analysis of DEGs from the two key modules revealed that the blue module was predominantly enriched in the ribosome (q-value = 0.009), carbon metabolism (q-value = 0.068), and biosynthesis of secondary metabolites (q-value = 0.104) (Fig. 6D and Table S10). Both carbon metabolism and biosynthesis of secondary metabolites were closely related to ethyl ester biosynthesis. In contrast, 14 pathways in the turquoise module were significantly enriched (Fig. 6E and Table S10). Notably, biosynthesis of secondary metabolites (q-value <0.001), carbon metabolism (q-value <0.001), pyruvate metabolism (q-value <0.05), and glycolysis/gluconeogenesis (q-value <0.05) were closely linked to the biosynthesis of ethyl ester. These findings indicate that the DEGs in both modules play vital roles in the synthesis and metabolism of ethyl esters.

To identify the key genes within the co-expression network, we filtered the eigengene pairs in the two key modules using a weight threshold of ≥0.4. (Wang et al., 2023). As a result, 13,910 and 120,751 linear pairs were identified in the blue and turquoise modules, respectively (Table S11–13).

The linear pairs selected from the blue module included 21 transcription factors (FC ≥ 1.2 in L-EP vs H-EP or L-SP vs H-SP) and 7 structural genes (FC ≥ 1.5 in L-EP vs H-EP or L-SP vs H-SP). Similarly, 41 transcription factors (FC ≥ 1.2 in L-EP vs H-EP or L-SP vs H-SP) and 40 structural genes (FC ≥ 1.5 in L-EP vs H-EP or L-SP vs H-SP) were identified in the turquoise module (Table S14). Among these structural genes, the expression of *ALD4*, *ACS1*, *ALD6*, *SOL3*, *GPD2*, and *GPP1* was found to potentially reduce ethyl ester levels in wine under high sugar stress. It is, therefore, proposed that these genes may play a pivotal role in regulating ethyl ester accumulation under varying sugar stress conditions. 8 key transcription factors, potentially regulating genes in the ethyl ester biosynthesis pathway, were identified through the Yeastract database (www.yeastract.com) and current research (Fig. 6F and G, Table S15) (Du et al., 2022; Haugen et al., 2004; Hlynialuk et al., 2008; Miles et al., 2013; Srinivasan et al., 2020; Vyas et al., 2005; Zhang et al., 2009; Zhang et al., 2016). Additionally, all kME values for these key transcription factors exceeded 0.85 (Table S15). These findings indicate that these transcription factors likely play a crucial role in ethyl ester accumulation under varying sugar stress conditions.

## 4 Conclusion

This study represents the first comprehensive investigation into the sensory profiles, ethyl ester biosynthesis, and the molecular mechanisms underlying the reduction of fruity aromas under sugar stress conditions during sweet wine fermentation. Our findings demonstrate that high initial sugar concentrations significantly reduce the overall aroma intensity, particularly the fruity notes, in sweet wines. Wines fermented with an initial sugar concentration of 204 g/L exhibited more pronounced aromas of apple, pineapple, peach, and passion fruit, which correlated with higher levels of phenethyl acetate, ethyl butanoate, ethyl hexanoate, ethyl octanoate, ethyl decanoate, and 2-phenylethyl alcohol. In contrast, wines fermented with an initial sugar concentration of 316 g/L displayed weakened fruity aromas, which were associated with lower concentrations of ethyl esters. This study is the first to systematically examine the evolution of ethyl esters during sweet wine fermentation under varying sugar stress conditions. It provides a deeper understanding of their potential impact on the overall flavor profile of the wine and helps elucidate the reasons behind the variations in ethyl ester accumulation under different sugar stress conditions. Transcriptomic analysis revealed that at higher initial sugar concentrations, *S. cerevisiae* upregulated the expression of low-affinity sugar transporter genes and downregulated high-affinity sugar transporter genes. Additionally, most genes involved in the glycolytic pathway, pentose phosphate pathway, and glycerol synthesis were upregulated, while those related to the TCA cycle and electron transport chain were downregulated, likely reflecting an adaptive response to hyperosmotic stress. WGCNA identified 14 sugar stress–responsive hub genes associated with ethyl ester biosynthesis, including six structural genes and eight transcription factors. These findings provide new insights into the molecular mechanisms that drive the reduction of fruity aromas under sugar stress during sweet wine fermentation and offer a theoretical basis for improving fruity aroma retention in sweet wines produced through high-sugar fermentations.

## CRediT authorship contribution statement

**Ruyi Li:** Writing – original draft, Visualization, Methodology, Investigation, Formal analysis. **Wenzhe Tong:** Methodology, Investigation. **You Liu:** Investigation. **Qian Ge:** Writing – review & editing. **Xiaoyu Xu:** Investigation. **Keji Yu:** Writing – review & editing, Conceptualization. **Wenyu Shi:** Writing – review & editing. **Haibin Mu:** Writing – review & editing. **Guoliang Yan:** Writing – review & editing. **Changqing Duan:** Writing – review & editing, Funding acquisition, Conceptualization. **Yibin Lan:** Writing – review & editing, Supervision, Project administration, Funding acquisition, Conceptualization.

## Declaration of competing interest

The authors declare that they have no known competing financial interests or personal relationships that could have appeared to influence the work reported in this paper.

The following are the supplementary data related to this article.

Supplementary Material 1 Supplementary Figures S1–S5 and Tables S1–S4. Supplementary material 1

Supplementary Material 2 Supplementary Tables S5–S15. Supplementary material 2

Supplementary data to this article can be found online at https://doi.org/10.1016/j.fochx.2026.103580.

## Data Availability

Data will be made available on request.

## References

[bb0005] Antalick G., Šuklje K., Blackman J.W., Meeks C., Deloire A., Schmidtke L.M. (2015). Influence of grape composition on red wine Ester profile: Comparison between cabernet sauvignon and shiraz cultivars from Australian warm climate. Journal of Agricultural and Food Chemistry.

[bb0010] Baldwin J.E., Krebs H. (1981). The evolution of metabolic cycles. Nature.

[bb0015] Başkan K.S., Tütem E., Akyüz E., Özen S., Apak R. (2016). Spectrophotometric total reducing sugars assay based on cupric reduction. Talanta.

[bb0020] Chevtzoff C., Yoboue E.D., Galinier A., Casteilla L., Daignan-Fornier B., Rigoulet M., Devin A. (2010). Reactive oxygen species-mediated regulation of mitochondrial biogenesis in the yeast *Saccharomyces cerevisiae**. Journal of Biological Chemistry.

[bb0025] Crabtree H.G. (1929). Observations on the carbohydrate metabolism of tumours. Biochemical Journal.

[bb0030] Dannenmaier S., Stiller S.B., Morgenstern M., Lübbert P., Oeljeklaus S., Wiedemann N., Warscheid B. (2018). Complete native stable isotope labeling by amino acids of *Saccharomyces cerevisiae* for global proteomic analysis. Analytical Chemistry.

[bb0035] Deng H., Du Z.-D., Lu S.-R., Wang Z.-Y., He X.-P. (2023). Regulation of *Cat8* in energy metabolic balance and glucose tolerance in *Saccharomyces cerevisiae*. Applied Microbiology and Biotechnology.

[bb0040] Ding R.-R., Che X.-K., Shen Z., Zhang Y.-H. (2021). Metabolome and transcriptome profiling provide insights into green apple peel reveals light- and UV-B-responsive pathway in anthocyanins accumulation. BMC Plant Biology.

[bb0045] Du Z.-D., Deng H., Cheng Y.-F., Zhai Z.-G., Guo X.-N., Wang Z.-Y., He X.-P. (2022). *Cat8* response to nutritional changes and interaction with ehrlich pathway related factors. Frontiers in Microbiology.

[bb0050] Duncan J.D., Setati M.E., Divol B. (2023). Redox cofactor metabolism in *Saccharomyces cerevisiae* and its impact on the production of alcoholic fermentation end-products. Food Research International.

[bb0055] Englezos V., Rantsiou K., Cravero F., Torchio F., Pollon M., Fracassetti D., Ortiz-Julien A., Gerbi V., Rolle L., Cocolin L. (2018). Volatile profile of white wines fermented with sequential inoculation of *Starmerella bacillaris* and *Saccharomyces cerevisiae*. Food Chemistry.

[bb0060] Erasmus D.J., van der Merwe G.K., van Vuuren H.J.J. (2003). Genome-wide expression analyses: Metabolic adaptation of *Saccharomyces cerevisiae* to high sugar stress. FEMS Yeast Research.

[bb0065] Ferreira V., López R., Cacho J.F. (2000). Quantitative determination of the odorants of young red wines from different grape varieties. Journal of the Science of Food and Agriculture.

[bb0070] Franco M., Peinado R.A., Medina M., Moreno J. (2004). Off-vine grape drying effect on volatile compounds and aromatic series in must from Pedro Xim’enez grape variety. J. Agric. Food Chemistry.

[bb0075] García R., Bermejo C., Grau C., Pérez R., Rodríguez-Peña J.M., Francois J., Arroyo J. (2004). The global transcriptional response to transient cell wall damage in *Saccharomyces cerevisiae* and its regulation by the cell integrity signaling pathway. Journal of Biological Chemistry.

[bb0080] Ge S., Xie Y., Ding K., Xu S.-Q., Xu H.-S., Chang X., Li H., Wang R.-R., Luo Z.-S., Shan Y., Ding S.-H. (2024). The combination of metabolome and transcriptome clarifies the inhibition of the alternaria toxin accumulation by methyl ferulate. Food Chemistry.

[bb0085] Gómez García-Carpintero E., Gómez Gallego M.A., Sánchez-Palomo E., González Viñas M.A. (2012). Impact of alternative technique to ageing using oak chips in alcoholic or in malolactic fermentation on volatile and sensory composition of red wines. Food Chemistry.

[bb0090] Guan N.-Z., Li J.-H., Shin H.-D., Du G.-C., Chen J., Liu L. (2017). Microbial response to environmental stresses: From fundamental mechanisms to practical applications. Applied Microbiology and Biotechnology.

[bb0095] Guth H. (1997). Quantitation and sensory studies of character impact odorants of different white wine varieties. Journal of Agricultural and Food Chemistry.

[bb0100] Han X., Wang Y., Lu H.-C., Yang H.-Y., Li H.-Q., Gao X.-T., Pei X.-X., He F., Duan C.-Q., Wang J. (2022). The combined influence of rootstock and vintage climate on the grape and wine flavonoids of *Vitis vinifera* L. cv. Cabernet Sauvignon in eastern China. Frontiers in Plant Science.

[bb0105] Haugen A.C., Kelley R., Collins J.B., Tucker C.J., Deng C., Afshari C.A., Van Houten B. (2004). Integrating phenotypic and expression profiles to map arsenic-response networks. Genome Biology.

[bb0110] Heit C., Martin S.J., Yang F., Inglis D.L. (2018). Osmoadaptation of wine yeast (*Saccharomyces cerevisiae*) during Icewine fermentation leads to high levels of acetic acid. Journal of Applied Microbiology.

[bb0115] Hlynialuk C., Schierholtz R., Vernooy A., Van Der Merwe G. (2008). *Nsf1*/*Ypl230w* participates in transcriptional activation during non-fermentative growth and in response to salt stress in *Saccharomyces cerevisiae*. Microbiology.

[bb0120] Houtman A.C., Marais J., Du Plessis C.S. (1980). Factors affecting the reproducibility of fermentation of grape juice and of the aroma composition of wines*.* 1. Grape maturity, sugar, inoculum concentration, aeration, juice turbidity and ergosterol. Vitis.

[bb0125] Jiménez-Martí E., Zuzuarregui A., Gomar-Alba M., Gutiérrez D., Gil C., del Olmo M. (2011). Molecular response of *Saccharomyces cerevisiae* wine and laboratory strains to high sugar stress conditions. International Journal of Food Microbiology.

[bb0130] Krüger A., Grüning N.M., Wamelink M.M.C., Kerick M., Kirpy A., Parkhomchuk D., Ralser M. (2011). The pentose phosphate pathway is a metabolic redox sensor and regulates transcription during the antioxidant response. Antioxidants & Redox Signaling.

[bb0135] Lan Y.-B., Xiang X.-F., Qian X., Wang J.-M., Ling M.-Q., Zhu B.-Q., Duan C.-Q. (2019). Characterization and differentiation of key odor-active compounds of ‘Beibinghong’ icewine and dry wine by gas chromatography-olfactometry and aroma reconstitution. Food Chemistry.

[bb0140] Langfelder P., Horvath S. (2008). WGCNA: An R package for weighted correlation network analysis. BMC Bioinformatics.

[bb0145] Lee P.-R., Ong Y.-L., Yu B., Curran P., Liu S.-Q. (2010). Profile of volatile compounds during papaya juice fermentation by a mixed culture of *Saccharomyces cerevisiae* and *Williopsis saturnus*. Food Microbiology.

[bb0150] Lee S.-W., Oh M.-K. (2016). Improved production of N-acetylglucosamine in *Saccharomyces cerevisiae* by reducing glycolytic flux. Biotechnology and Bioengineering.

[bb0155] Lemus M.R., Roussarie E., Hammad N., Mougeolle A., Ransac S., Issa R., Devin A. (2018). The role of glycolysis-derived hexose phosphates in the induction of the Crabtree effect. Journal of Biological Chemistry.

[bb0160] Li H. (2006).

[bb0165] Li Q., He F., Zhu B.-Q., Liu B., Sun R.-Z., Duan C.-Q., Wang J. (2014). Comparison of distinct transcriptional expression patterns of flavonoid biosynthesis in cabernet sauvignon grapes from east and West China. Plant Physiology and Biochemistry.

[bb0170] Ling M.-Q., Qi M.-Y., Li S.-Y., Shi Y., Pan Q.-H., Cheng C.-F., Duan C.-Q. (2022). The influence of polyphenol supplementation on ester formation during red wine alcoholic fermentation. Food Chemistry..

[bb0175] Liu C.-H., Chen Z.-H., Chen J., Wang S., Li J., Mao X.-Z. (2023). Transcriptome analysis reveals the potential mechanism of carotenoids change in hepatopancreas under low-temperature storage from swimming crab (*Portunus trituberculatus*). Food Chemistry.

[bb0180] Liu S.-X., Laaksonen O., Marsol-Vall A., Zhu B.-Q., Yang B.-R. (2020). Comparison of volatile composition between alcoholic bilberry beverages fermented with non-*Saccharomyces* yeasts and dynamic changes in volatile compounds during fermentation. Journal of Agricultural and Food Chemistry.

[bb0185] Liu S.-X., Lou Y., Li Y.-X., Zhao Y., Laaksonen O., Li P., Zhang J.-J., Battino M., Yang B.-R., Gu Q. (2023). Aroma characteristics of volatile compounds brought by variations in microbes in winemaking. Food Chemistry.

[bb0190] Liu X., Qin L., Yu J., Sun W.-T., Xu J.-H., Li C. (2023). Real-time monitoring of subcellular states with genetically encoded redox biosensor system (RBS) in yeast cell factories. Biosensors and Bioelectronics.

[bb0195] Livak K.J., Schmittgen T.D. (2001). Analysis of relative gene expression data using real-time quantitative PCR and the 2(-Delta Delta C(T)) method. Methods.

[bb0200] Longo R., Carew A., Sawyer S., Kemp B., Kerslake F. (2021). A review on the aroma composition of *Vitis vinifera* L. pinot noir wines: Origins and influencing factors. Critical Reviews in Food Science and Nutrition.

[bb0205] Lu H.-C., Tian M.-B., Han X., Shi N., Li H.-Q., Cheng C.-F., Wang J. (2023). The key role of vineyard parcel in shaping flavonoid profiles and color characteristics of cabernet sauvignon wines combined with the influence of harvest ripeness, vintage and bottle aging. Food Chemistry: X.

[bb0210] Lu Y., Chan L.-J., Li X., Liu S.-Q. (2018). Effects of sugar concentration on mango wine composition fermented by *Saccharomyces cerevisiae* MERIT.Ferm. International Journal of Food Science and Technology.

[bb0215] Maier A., Völker B., Boles E., Fuhrmann G.F. (2002). Characterisation of glucose transport in *Saccharomyces cerevisiae* with plasma membrane vesicles (countertransport) and intact cells (initial uptake) with single *Hxt1*, *Hxt2*, *Hxt3*, *Hxt4*, *Hxt6*, *Hxt7* or *Gal2* transporters. FEMS Yeast Research.

[bb0220] Malina C., Yu R., Björkeroth J., Kerkhoven E.J., Nielsen J. (2021). Adaptations in metabolism and protein translation give rise to the Crabtree effect in yeast. Proceedings of the National Academy of Sciences.

[bb0225] Martín-García F.J., Palacios-Fernández S., López de Lerma N., García-Martínez T., Mauricio J.C., Peinado R.A. (2023). The effect of yeast, sugar and sulfur dioxide on the volatile compounds in wine. Fermentation.

[bb0230] Miles S., Li L., Davison J., Breeden L.L. (2013). *Xbp1* directs global repression of budding yeast transcription during the transition to quiescence and is important for the longevity and reversibility of the quiescent state. PLoS Genetics.

[bb0235] Moyano L., Zea L., Moreno J., Medina M. (2002). Analytical study of aromatic series in sherry wines subjected to biological aging. Journal of Agricultural and Food Chemistry.

[bb0240] Noti O., Vaudano E., Pessione E., Garcia-Moruno E. (2015). Short-term response of different *Saccharomyces cerevisiae* strains to hyperosmotic stress caused by inoculation in grape must: RT-qPCR study and metabolite analysis. Food Microbiology.

[bb0245] Peinado R.A., Mauricio J.C., Moreno J. (2006). Aromatic series in sherry wines with gluconic acid subjected to different biological aging conditions by Saccharomyces cerevisiae var. Capensis. Food Chemistry.

[bb0250] Peinado R.A., Moreno J., Bueno J.E., Moreno J.A., Mauricio J.C. (2004). Comparative study of aromatic compounds in two young white wines subjected to pre-fermentative cryomaceration. Food Chemistry.

[bb0255] Peng C.-T., Wen Y., Tao Y.-S., Lan Y.-Y. (2013). Modulati’ng the formation of Meili wine aroma by prefermentative freezing process. Journal of Agricultural and Food Chemistry.

[bb0260] Perl A., Hanczko R., Telarico T., Oaks Z., Landas S. (2011). Oxidative stress, inflammation and carcinogenesis are controlled through the pentose phosphate pathway by transaldolase. Trends in Molecular Medicine.

[bb0265] Pigeau G.M., Inglis D.L. (2005). Upregulation of *ALD3* and *GPD1* in *Saccharomyces cerevisiae* during icewine fermentation. Journal of Applied Microbiology.

[bb0270] Puria R., Mannan M.A., Chopra-Dewasthaly R., Ganesan K. (2009). Critical role of *RPI1* in the stress tolerance of yeast during ethanolic fermentation. FEMS Yeast Research..

[bb0275] Reboredo-Rodríguez P., González-Barreiro C., Rial-Otero R., Cancho-Grande B., Simal-Gándara J. (2015). Effects of sugar concentration processes in grapes and wine aging on aroma compounds of sweet wines—A review. Critical Reviews in Food Science and Nutrition.

[bb0280] Ríos-Reina R., Segura-Borrego M.P., Morales M.L., Callejón R.M. (2020). Characterization of the aroma profile and key odorants of the Spanish PDO wine vinegars. Food Chemistry.

[bb0285] Saerens S.M.G., Delvaux F.R., Verstrepen K.J., Thevelein J.M. (2010). Production and biological function of volatile esters in *Saccharomyces cerevisiae*. Microbial Biotechnology.

[bb0290] Srinivasan R., Walvekar A.S., Rashida Z., Seshasayee A., Laxman S. (2020). Genome-scale reconstruction of *Gcn4*/*ATF4* networks driving a growth program. PLoS Genetics.

[bb0295] Sumby K.M., Grbin P.R., Jiranek V. (2010). Microbial modulation of aromatic esters in wine: Current knowledge and future prospects. Food Chemistry.

[bb0300] Swiegers, J. H., & Pretorius, I. S. (2005). Yeast modulation of wine flavor. Advances In Applied Microbiology (vol. 57, pp. 131–175). Elsevier. doi:10.1016/s0065-2164(05)57005-9. 16002012

[bb0305] Tao Y.-S., Zhang L. (2010). Intensity prediction of typical aroma characters of cabernet sauvignon wine in Changli County (China). LWT-Food Science and Technology.

[bb0310] Tatebayashi K., Yamamoto K., Tomida T., Nishimura A., Takayama T., Oyama M., Kozuka-Hata H., Adachi-Akahane S., Tokunaga Y., Saito H. (2020). Osmostress enhances activating phosphorylation of Hog1 MAP kinase by mono-phosphorylated Pbs2 MAP2K. EMBO Journal.

[bb0315] Toledano M.B., Delaunay A., Biteau B., Spector D., Azevedo D. (2003). Yeast stress responses.

[bb0320] Verduyn C., Zomerdijk T.P.L., van Dijken J.P., Scheffers W.A. (1984). Continuous measurement of ethanol production by aerobic yeast suspensions with an enzyme electrode. Applied Microbiology and Biotechnology.

[bb0325] Vyas V.K., Berkey C.D., Miyao T., Carlson M. (2005). Repressors *Nrg1* and *Nrg2* regulate a set of stress-responsive genes in *Saccharomyces cerevisiae*. Eukaryotic Cell.

[bb0330] Wang R.-Q., Wang Y.-T., Yao W.-J., Ge W.-G., Jiang T.-B., Zhou B.-R. (2023). Transcriptome sequencing and WGCNA reveal key genes in response to leaf blight in poplar. International Journal of Molecular Sciences.

[bb0335] Zea L., Moyano L., Moreno J.A., Medina M. (2007). Aroma series as fingerprints for biological ageing in fino sherry-type wines. Journal of the Science of Food and Agriculture.

[bb0340] Chen Y.-R., Yang Y.-L., Cai W.-Q., Zeng J.-L., Liu N., Wan Y., Fu G.-M. (2023). Research progress of anti-environmental factor stress mechanism and anti-stress tolerance way of *Saccharomyces cerevisiae* during the brewing process. Critical Reviews in Food Science and Nutrition.

[bb0345] Zhang B.-B., Hao L.-F., Zhang J., Feng J.-Z., Wang C., Zhang J.-F. (2024). Integration of transcriptome, volatile and non-volatile metabolite profile reveals characteristic aroma formation in *Toona sinensis*. Food Chemistry.

[bb0350] Zhang B.-Q., Tang C., Yang D.-Q., Liu H., Xue J., Duan C.-Q., Yan G.-L. (2022). Effects of three indigenous non-*Saccharomyces* yeasts and their pairwise combinations in co-fermentation with *Saccharomyces cerevisiae* on volatile compounds of petit manseng wines. Food Chemistry.

[bb0355] Zhang C., Li Z.-Q., Zhang X.-H., Yuan L., Dai H.-P., Xiao W. (2016). Transcriptomic profiling of chemical exposure reveals roles of *Yap1* in protecting yeast cells from oxidative and other types of stresses. Yeast.

[bb0360] Zhang N.-S., Wu J., Oliver S.G. (2009). *Gis1* is required for transcriptional reprogramming of carbon metabolism and the stress response during transition into stationary phase in yeast. *Microbiology*, *155*(5), 1690–1698. Microbiology.

[bb0365] Zhou J., Guo H., Liu L.-K., Hao S.-L., Guo Z., Zhang F.-P., Gao Y., Wang Z., Zhang W.-W. (2021). Construction of co-expression modules related to survival by WGCNA and identification of potential prognostic biomarkers in glioblastoma. Journal of Cellular and Molecular Medicine.

