# Supplementary material for: Sugar stress attenuates fruity aroma in sweet wine by suppressing ethyl ester biosynthesis: Insights from integrated sensory, metabolome, and transcriptomic analyses
Source: Food Chem X. 2026 Jan 30;34:103580. doi: 10.1016/j.fochx.2026.103580 (PMC12926643; doi:10.1016/j.fochx.2026.103580)
Supplement: Supplementary Material 1 — Supplementary Figures S1–S5 and Tables S1–S4. [file mmc1.docx]

**Supplementary materials**

**Figure captions**

**Fig. S1** RT-qPCR validation of 12 DEGs in the sugar consumption levels of 120 g/L and 180 g/L, respectively. (A) Correlation analysis of gRT-PCR and RNA-seq. (B) Linear regression of RNA-seq and qRT-PCR data that are expressed as a log2 fold change.

**Fig. S2** The correlation heat map (A) and principal component analysis (PCA) (B) of 18 samples based on their gene expression levels. Volcano plot of DEGs in L-EP vs M-EP (C), L-EP vs H-EP (D), L-SP vs M-SP (E), and L-SP vs H-SP (F)

**Fig. S3** Expression of the structural genes of the glycolysis pathway in *S. cerevisiae*. Asterisks indicate DEGs (fold change (FC) ≥ 1.5 and *P* < 0.05).

**Fig. S4** Expression of the structural genes of pentose phosphate pathway in *S. cerevisiae*. Asterisks indicate DEGs (fold change (FC) ≥ 1.5 and *P* < 0.05).

**Fig. S5** Expression of the structural genes of TCA cycle and electron transport chain in *S. cerevisiae.* Asterisks indicate DEGs (fold change (FC) ≥ 1.5 and *P* < 0.05).

**Fig. S1** qRT-PCR validation of 12 DEGs in the sugar consumption levels of 120 g/L and 180 g/L, respectively. (A) Correlation analysis of gRT-PCR and RNA-seq. (B) Linear regression of RNA-seq and qRT-PCR data that are expressed as a log2 fold change.

**Fig. S2** The correlation heat map (A) and principal component analysis (PCA) (B) of 18 samples based on their gene expression levels. Volcano plot of DEGs in L-EP vs M-EP (C), L-EP vs H-EP (D), L-SP vs M-SP (E), and L-SP vs H-SP (F)

**Fig. S3** Expression of the structural genes of the glycolysis pathway in *S. cerevisiae*. Asterisks indicate DEGs (fold change (FC) ≥ 1.5 and *P* < 0.

**Fig. S4** Expression of the structural genes of pentose phosphate pathway in *S. cerevisiae*. Asterisks indicate DEGs (fold change (FC) ≥ 1.5 and *P* < 0.05).

**Fig. S5** Expression of the structural genes of TCA cycle and electron transport chain in *S. cerevisiae.* Asterisks indicate DEGs (fold change (FC) ≥ 1.5 and *P* < 0.05).

**Table captions**

**Table S1** Details of the standards used for qualitative and quantitative analysis of aroma compounds.

**Table S2** Aroma attributes used for training sessions and preparation of reference standards.

**Table S3** List of RT-qPCR primer sequences used.

**Table S4** Overview of the transcriptome sequencing dataset.

**Table S5** The common DEGs in L-EP vs M-EP, L-EP vs H-EP, L-SP vs M-SP, and L-SP vs H-SP.

**Table S6** KEGG enrichment analysis of DEGs in L-EP vs M-EP.

**Table S7** KEGG enrichment analysis of DEGs in L-EP vs H-EP.

**Table S8** KEGG enrichment analysis of DEGs in L-SP vs M-SP.

**Table S9** KEGG enrichment analysis of DEGs in L-SP vs H-SP.

**Table S10** KEGG enrichment analysis of DEGs in blue and turquoise module.

**Table S11** List of genes table.

**Table S12** Linear pairs of eigengenes with weight≥0.4 in the blue module.

**Table S13** Linear pairs of eigengenes with weight≥0.4 in the turquoise module.

**Table S14** Important genes in the blue and turquoise modules.

**Table S15** Hub genes in the blue and turquoise modules.

**Table S1** Details of the standards used for qualitative and quantitative analysis of aroma compounds

| Compounds | CAS | Manufacturer^a^ | Product code | Purity^b^ | RI^c^ | Quantified ions (*m/z*) | Linear range  (µg/L)^d^ | R^2^  value |
| --- | --- | --- | --- | --- | --- | --- | --- | --- |
| Aroma compounds |  |  |  |  |  |  |  |  |
| *Acetate esters* |  |  |  |  |  |  |  |  |
| Isobutyl acetate | 110190 | Sigma | 537470 | 99% | 990.1 | 43 | 1.5-240 | 0.999 |
| Isoamyl acetate | 19329896 | TCI | A0033 | 98% | 1126.2 | 43 | 34.35-2748 | 0.996 |
| Hexyl acetate | 142927 | Sigma | 108154 | 99.7% | 1258.1 | 43 | 1.2-38.4 | 0.994 |
| Heptyl acetate | 112061 | Sigma | W254703 | 98% | 1363.6 | 43 | 0.15-6.08 | 0.990 |
| Phenethyl acetate | 103457 | Sigma | 290580 | 99% | 1813.9 | 104 | 2.2-268.8 | 0.997 |
| Ethyl acetate | 141786 | Sigma | 270989 | 99.8% | 898.8 | 43 | 1684.3-134744 | 0.993 |
| *Ethyl esters* |  |  |  |  |  |  |  |  |
| Ethyl butanoate | 105544 | Sigma | E15701 | 99% | 1042.7 | 71 | 11.67-933.6 | 0.998 |
| Ethyl hexanoate | 123660 | Sigma | 148962 | 99% | 1224.1 | 88 | 29.09-2327.2 | 0.992 |
| Ethyl (*E*)-3-hexenoate | 2396830 | Sigma | 340294 | 97% | 1310.1 | 41 | 0.2-8 | 0.994 |
| Ethyl octanoate | 106321 | Sigma | W244902 | 99% | 1424.1 | 88 | 41-3280 | 0.997 |
| Ethyl decanoate | 110383 | Sigma | W243205 | 99% | 1626.5 | 88 | 17-2720 | 0.993 |
| Ethyl dodecanoate | 106332 | Sigma | 61630 | 98% | 1837.9 | 88 | 27.06-270.6 | 0.996 |
| Ethyl tetradecanoate | 124061 |  |  |  | 2027.8 | 88 | Ethyl hexadecanoate | Ethyl hexadecanoate |
| Ethyl hexadecanoate | 628977 | Sigma | P9009 | 99% | 2215.3 | 88 | 10.66-319.8 | 0.993 |
| *Other esters* |  |  |  |  |  |  |  |  |
| Methyl hexanoate | 106707 | Sigma | 259942 | 99% | 1191.7 | 74 | 0.34-13.6 | 0.998 |
| Methyl octanoate | 111115 | Sigma | 260673 | 99% | 1365.1 | 74 | 0.66-26.4 | 0.992 |
| Isobutyl octanoate | 5461063 | TCI | O0032 | 99% | 1557.4 | 57 | 1.62-25.92 | 0.986 |
| Methyl decanoate | 110429 |  |  |  | 1583.1 | 74 | Ethyl dodecanoate | Ethyl dodecanoate |
| *Higher alcohols* |  |  |  |  |  |  |  |  |
| 1-octanol | 111875 | Sigma | 297887 | 99% | 1542.5 | 56 | 0.5-79.36 | 0.997 |
| Isopentanol | 123513 | Sigma | 309435 | 99% | 1185.7 | 55 | 3983.68-478041.6 | 0.995 |
| 1-propanol | 71238 | Sigma | 34871 | 99.9% | 1057 | 31 | 858.8-137408 | 0.999 |
| 1-butanol | 71363 | Sigma | 34867 | 99.7% | 1151.0 | 56 | 34.88-6796 | 0.997 |
| Isobutanol | 78831 | Sigma | 538132 | 99.5% | 1073.6 | 43 | 1588.48-190617.6 | 0.999 |
| 4-methyl-1-pentanol | 626891 | Sigma | M66951 | 97% | 1300.2 | 56 | 1.36-217.6 | 0.999 |
| 1-decanol | 112301 | Sigma | 150584 | 98% | 1744.7 | 70 | 0.192-30.72 | 0.991 |
| 2-Phenylethyl alcohol | 60128 | Sigma | 77861 | 99% | 1905.9 | 91 | 2409.92-289190.4 | 0.998 |
| *C6 alcohols* |  |  |  |  |  |  |  |  |
| 1-hexanol | 111273 | Sigma | H13303 | 98% | 1338.7 | 56 | 50.06-8009.6 | 0.996 |
| (*E*)-2-hexen-1-ol | 92895 | Sigma | 132667 | 96% | 1409 | 57 | 1.26-201.6 | 0.999 |
| (Z)-3-hexen-1-ol | 928961 | Sigma | H12900 | 98% | 1387 | 67 | 3.18-508.8 | 0.999 |
| (*E*)-3-hexen-1-ol | 928972 | Sigma | 224715 | 98% | 1350.9 | 41 | 1.88-112.8 | 0.994 |
| *Volatile fatty acids* |  |  |  |  |  |  |  |  |
| Hexanoic acid | 142621 | Sigma | 153745 | 99% | 1835.7 | 60 | 156.8-7840 | 0.998 |
| Octanoic acid | 124072 | Sigma | C2875 | 99% | 2041.8 | 60 | 129.76-6488 | 0.997 |
| Decanoic acid | 334485 | Sigma | C1875 | 98% | 2234.3 | 60 | 61.28-3064 | 0.995 |
| Butanoic acid | 107926 | Sigma | W222100 | 99% | 1618.7 | 60 | 220-5500 | 1.000 |
| Isopentanoic acid | 503742 | Sigma | 129542 | 99% | 1696.2 | 60 | 92.20-2305 | 0.995 |
| Isobutanoic acid | 79312 | Sigma | W222208 | 99% | 1597.4 | 43 | 122.88-3072 | 0.991 |
| *Others* |  |  |  |  |  |  |  |  |
| Citronellol | 106229 | Sigma | W230901 | 95% | 1753.9 | 69 | 0.43-12.90 | 0.999 |
| *α*-terpineol | 98555 | Sigma | 04899 | analytical standard | 1684.8 | 59 | 0.41-12.3 | 0.996 |
| Linalool | 78706 | Sigma | L2602 | 97% | 1547 | 71 | 0.44-13.2 | 0.999 |
| Styrene | 100425 | TCI | S0095 | 99% | 1252.6 | 104 | 0.34-27.2 | 0.990 |

^a^ Sigma: Sigma-Aldrich (St. Louis, MO, USA); TCI: TCI (Shanghai) Development Co., Ltd. (Shanghai, China);

^b^ Greater than or equal to the purity of the standard.

^c^ Retention indices on HP-INNOWax column;

^d^ The semi-quantification of ethyl tetradecanoate and methyl decanoate was based on the calibration curves of the listed compound

Table S2 Aroma attributes used for training sessions and preparation of reference standards

| Descriptor | Odor reference |  | Reference standards for training ^a^ |
| --- | --- | --- | --- |
|  |  |  |  |
| Vegetal | Reference standard compounds obtained from Sigma-Aldrich |  | (*E*)-3-hexenol (400 mg/L) prepared in 50 mL model wine solution. |
| Floral | Reference standard compounds obtained from Sigma-Aldrich |  | Phenylethanol (5000 mg/L) and linalool (20 mg/L), prepared in 50 mL model wine solution |
| Apple | Natural product |  | Commercial apple |
| Pineapple | Natural product |  | Commercial pineapple |
| Honey | Solution obtained from Aipu Food Industry |  | 1 drop/50 mL distilled water |
| Peach | Natural product |  | Commercial peach |
| Citrus | Natural product |  | Commercial citrus |
| Passion fruit | Natural product |  | Commercial passion fruit |
| Kerosene | Solution obtained from Aipu Food Industry |  | Take 10μL of kerosene and add it to 50mL of model wine solution. |
| Chemical | Reference standard compounds obtained from Sigma-Aldrich |  | Ethyl acetate (0.5 mg/L) prepared in 50 mL model wine solution. |

^a^ Reference standards were prepared in the model wine solution (12% v/v ethanol, 2 g/L glucose, 7 g/L tartaric acid, pH 3.5).

Table S3 List of RT-qPCR primer sequences used

| Gene name | Forward (5'-> 3') | Reverse (5'->3') |
| --- | --- | --- |
| ALD4 | TGGAGCTGGGTGGTAAATCAC | ATAATGTTTTGCACGGCTTTTTTC |
| ALD6 | CGCCCATTTGGTCTTTGACG | AGCAGCCAATAGTTCGTCGT |
| ACS1 | GCTGCAAAGGATAAGGATGG | GCCTCAATTTCAGCGGTAGA |
| USV1 | ATTCATTCTCCCCGCCGTTT | CTTTGCCGCCATTCTTCGAG |
| GSM1 | TACGAATAACCGCCAGACCG | TACGGTGGGTATGATGGGGT |
| CAT8 | GTGTGCTGCTGTCGGATTTG | ATTCTCTGCCTCCAGTTCGC |
| HXT1 | AACAAATGCCCACCTGACCA | AATTCGCCCCAAGATGCAGT |
| FMP16 | TGGAACACGGCAAGAATCCA | ACCGTCATCTGGCCTGTTTT |
| YAP1 | AGCGAGCAACCGAAGAAGAA | CCGATTTTGGGCAGTCCTCT |
| GCN4 | TGCTTACAACCGCAAACAGC | GCACGTTTTAGAGCAGCAGG |
| SOL3 | TTGGTACTGTTGGGCTGTGG | TTGGAGGCTTGGGAGAATCG |
| NRG2 | AGCAAACCGACAAAAACCGT | GACCAGAAGTGGTGAACCCA |
| RPN4 | CCCCCACAACAAACCTACCA | CTATCGTTGGCCGTTGCTTC |
| XBP1 | GCCAAAGAGACGGACATCCA | TCGGGCTTGATGTTGTTCCA |
| GIS1 | TAGGCGGCGATAACGATGAC | GCCGTTCGGATTCATTGGTG |
| GPP1 | GTAAGCCTCACCCAGAACCA | TTACAGCCAGCAGCCTTACC |
| GPD2 | TGGGATGGGGTAACAATGCC | GCAACACCAGCGGATTCTTG |
| **Internal control** |  |  |
| ACT1 | GAAATGCAAACCGCTGCTCA | TACCGGCAGATTCCAAACCC |

Table S4 Overview of the transcriptome sequencing dataset

| Sample | Total Reads | Mapped Reads | Mapping ratio | %≥Q30 | GC(%) |
| --- | --- | --- | --- | --- | --- |
| A120-1 | 44,636,098 | 43,543,068 | 97.55% | 97.14 | 41.37 |
| A120-2 | 46,278,228 | 45,053,426 | 97.35% | 96.85 | 41.47 |
| A120-3 | 45,393,416 | 44,253,937 | 97.49% | 97.1 | 41.46 |
| A180-1 | 47,984,244 | 46,981,692 | 97.91% | 97.47 | 41.48 |
| A180-2 | 47,393,424 | 46,036,732 | 97.14% | 96.51 | 41.51 |
| A180-3 | 48,641,952 | 47,561,238 | 97.78% | 97.37 | 41.51 |
| B120-1 | 46,377,578 | 45,205,229 | 97.47% | 97.07 | 41.44 |
| B120-2 | 48,585,526 | 47,391,692 | 97.54% | 97.64 | 41.52 |
| B120-3 | 45,089,728 | 43,902,213 | 97.37% | 96.75 | 41.52 |
| B180-1 | 43,209,264 | 42,240,485 | 97.76% | 97.43 | 41.52 |
| B180-2 | 43,368,084 | 42,385,700 | 97.73% | 97.53 | 41.5 |
| B180-3 | 45,007,756 | 44,133,813 | 98.06% | 97.44 | 41.31 |
| C120-1 | 49,825,916 | 48,426,498 | 97.19% | 96.38 | 41.69 |
| C120-2 | 44,655,374 | 43,750,167 | 97.97% | 97.5 | 41.66 |
| C120-3 | 44,303,156 | 43,384,661 | 97.93% | 97.82 | 41.89 |
| C180-1 | 45,535,658 | 44,526,156 | 97.78% | 97.4 | 41.4 |
| C180-2 | 52,356,706 | 50,643,271 | 96.73% | 95.84 | 41.51 |
| C180-3 | 42,114,516 | 41,219,846 | 97.88% | 97.64 | 41.4 |
